# Clinical and neuroradiological spectrum of biallelic variants in *NOTCH3*

**DOI:** 10.1016/j.ebiom.2024.105297

**Published:** 2024-08-26

**Authors:** Pablo Iruzubieta, César Augusto Pinheiro Ferreira Alves, Aisha M. Al Shamsi, Gehad ElGhazali, Maha S. Zaki, Lorenzo Pinelli, Diego Lopergolo, Bernard P.H. Cho, Amy A. Jolly, Amna Al Futaisi, Fatema Al-Amrani, Jessica Galli, Elisa Fazzi, Katarina Vulin, Francisco Barajas-Olmos, Holger Hengel, Bayan Mohammed Aljamal, Vahideh Nasr, Farhad Assarzadegan, Michele Ragno, Luigi Trojano, Naomi Meave Ojeda, Arman Çakar, Silvia Bianchi, Francesca Pescini, Anna Poggesi, Amal Al Tenalji, Majid Aziz, Rahema Mohammad, Aziza Chedrawi, Nicola De Stefano, Giovanni Zifarelli, Ludger Schöls, Tobias B. Haack, Adriana Rebelo, Stephan Zuchner, Filiz Koc, Lyn R. Griffiths, Lorena Orozco, Karla García Helmes, Meisam Babaei, Peter Bauer, Won Chan Jeong, Ehsan Ghayoor Karimiani, Miriam Schmidts, Joseph G. Gleeson, Wendy K. Chung, Fowzan Sami Alkuraya, Bita Shalbafan, Hugh S. Markus, Henry Houlden, Reza Maroofian

**Affiliations:** a Department of Neurogenetics, UCL Institute of Neurology London Queen Square and National Hospital for Neurology and Neurosurgery, University College London, London, United Kingdom; b Department of Neurology, Donostia University Hospital, Biogipuzkoa Health Research Institute, Donostia-San Sebastián, Spain; c CIBERNED, Centro de Investigación Biomédica en Red en Enfermedades Neurodegenerativas-Instituto de Salud Carlos III (CIBER-CIBERNED-ISCIII), 28029, Madrid, Spain; d Neuroradiology Division, Department of Radiology, Boston Children's Hospital, Harvard Medical School, Boston, MA, USA; e Division of Neuroradiology, Department of Radiology, Children's Hospital of Philadelphia, 3401 Civic Center Blvd., Philadelphia, PA, 19104, USA; f Genetic Division, Paediatrics Department, Tawam Hospital, Al-Ain, United Arab Emirates; g Sheikh Khalifa Medical City, Purelab, Purehealth, Abu Dhabi, United Arab Emirates; h College of Medicine and Health Sciences, United Arab Emirates University, Al Ain, United Arab Emirates; i Clinical Genetics Department, Human Genetics and Genome Research Institute, National Research Centre, El-Tahrir Street, Dokki, Cairo, Egypt; j Neuroradiology Unit, Pediatric Neuroradiology Section, ASST SpedaliCivili, Brescia, Italy; k Department of Medicine, Surgery and Neurosciences, University of Siena, Siena, Italy; l UOC Neurologia e Malattie Neurometaboliche, Azienda Ospedaliero-Universitaria Senese, Siena, Italy; m Department of Clinical Neurosciences, University of Cambridge, United Kingdom; n Department of Child Health, College of Medicine and Health Sciences, Sultan Qaboos University, Oman; o Child Neurology and Psychiatry Unit, ASST SpedaliCivili of Brescia, Brescia, Italy; p Department of Clinical and Experimental Sciences, University of Brescia, Brescia, Italy; q Department of Medical and Laboratory Genetics, ERN-Ithaca Zagreb Center, Children's Hospital Zagreb, Zagreb, Croatia; r Centre of Excellence for Reproductive and Regenerative Medicine, Medical School University of Zagreb, Zagreb, Croatia; s Immunogenomics and Metabolic Diseases Laboratory, Instituto Nacional de Medicina Genómica, SS, CDMX, Mexico; t Department of Neurology and Hertie-Institute for Clinical Brain Research, University of Tübingen, 72076, Tübingen, Germany; u German Center of Neurodegenerative Diseases (DZNE), 72076, Tübingen, Germany; v Department of Translational Genomics, Centre for Genomic Medicine, King Faisal Specialist Hospital and Research Centre, Riyadh, 11211, Saudi Arabia; w Department of Neurology - Kermanshah Imam Reza (AS) Hospital Complex, Kermanshah University of Medical Sciences, Kermanshah, Iran; x Department of Neurology, Imam Hossein Hospital, Shahid Beheshti University of Medical Sciences(SBUMS), Tehran, Iran; y Pianeta Salute, Viale Assisi, 88, 63084, Villa Pigna, Ascoli Piceno, Italy; z Department of Psychology, University of Campania ‘Luigi Vanvitelli’, Viale Ellittico 31, 81100, Caserta, Italy; aa Rady Children's Institute for Genomic Medicine, University of California, San Diego, La Jolla, USA; ab Neuromuscular Unit, Department of Neurology, Istanbul Faculty of Medicine, Istanbul University, 34093, Istanbul, Turkey; ac Department of Emergency, Stroke Unit, Careggi University Hospital, Florence, Italy; ad Department of NEUROFARBA, University of Florence, Florence, Italy; ae Sheikh Khalifa Medical City, Department of Pediatric Neurology, Abu Dhabi, United Arab Emirates; af Department of Neurosciences, King Faisal Specialist Hospital and Research Center, Riyadh, Saudi Arabia; ag CENTOGENE GmbH, Am Strande 7, 18055, Rostock, Germany; ah Institute of Medical Genetics and Applied Genomics, University of Tübingen, Tübingen, Germany; ai Dr. John T. Macdonald Foundation Department of Human Genetics and John P. Hussman Institute for Human Genomics, University of Miami Miller School of Medicine, Miami, FL, USA; aj Department of Neurology, Faculty of Medicine, Cukurova University, Adana, Turkey; ak Genomics Research Centre, Centre for Genomics and Personalised Health, School of Biomedical Sciences, Queensland University of Technology, 60 Musk Ave, Kelvin Grove, QLD, 4059, Australia; al Department of Genetics, General Hospital - Dr. Aurelio Valdivieso, Oaxaca de Juárez, Oaxaca, Mexico; am Department of Pediatrics, North Khorasan University of Medical Sciences, Bojnurd, Iran; an 3billion, Seoul, South Korea; ao Genetics Section, Molecular and Clinical Sciences Research Institute, St. George's, University of London, London, United Kingdom; ap Pediatrics Genetics Division, Center for Pediatrics and Adolescent Medicine, Faculty of Medicine, Freiburg University, Mathildenstrasse 1, 79106, Freiburg, Germany; aq CIBSS-Centre for Integrative Biological Signalling Studies, University of Freiburg, Freiburg, Germany; ar Department of Pediatrics, Boston Children's Hospital and Harvard Medical School, USA; as Cellular and Molecular Endocrine Research Center, Shahid Beheshti University of Medical Sciences, Tehran, Iran

**Keywords:** NOTCH3, CADASIL, Leukoencephalopathy, Stroke, Neurodevelopmental disorders

## Abstract

**Background:**

*NOTCH3* encodes a transmembrane receptor critical for vascular smooth muscle cell function. *NOTCH3* variants are the leading cause of hereditary cerebral small vessel disease (SVD). While monoallelic cysteine-involving missense variants in *NOTCH3* are well-studied in cerebral autosomal dominant arteriopathy with subcortical infarcts and leukoencephalopathy (CADASIL), patients with biallelic variants in *NOTCH3* are extremely rare and not well characterised.

**Methods:**

In this study, we present clinical and genetic data from 25 patients with biallelic *NOTCH3* variants and conduct a literature review of another 25 cases (50 patients in total). Brain magnetic resonance imaging (MRI) were analysed by expert neuroradiologists to better understand the phenotype associated with biallelic *NOTCH3* variants.

**Findings:**

Our systematic analyses verified distinct genotype-phenotype correlations for the two types of biallelic variants in *NOTCH3*. Biallelic loss-of-function variants (26 patients) lead to a neurodevelopmental disorder characterised by spasticity, childhood-onset stroke, and periatrial white matter volume loss resembling periventricular leukomalacia. Conversely, patients with biallelic cysteine-involving missense variants (24 patients) fall within CADASIL spectrum phenotype with early adulthood onset stroke, dementia, and deep white matter lesions without significant volume loss. White matter lesion volume is comparable between patients with biallelic cysteine-involving missense variants and individuals with CADASIL. Notably, monoallelic carriers of loss-of-function variants are predominantly asymptomatic, with only a few cases reporting nonspecific headaches.

**Interpretation:**

We propose a *NOTCH3-*SVD classification depending on dosage and variant type. This study not only expands our knowledge of biallelic *NOTCH3* variants but also provides valuable insight into the underlying mechanisms of the disease, contributing to a more comprehensive understanding of *NOTCH3*-related SVD.

**Funding:**

The 10.13039/100010269Wellcome Trust, the MRC.

**Research in context**

**Evidence before this study**

*NOTCH3* encodes for a transmembrane protein essential for brain small blood vessels. *NOTCH3* cysteine-involving heterozygous variants are well-known to cause cerebral autosomal dominant arteriopathy with subcortical infarcts and leukoencephalopathy (CADASIL) a disease characterised by migraine, dementia, and stroke. Additionally, very few case reports showing patients with biallelic variants have been described in the literature. Before initiating this study, we thoroughly considered existing evidence related to *NOTCH3* and its association with neurological disorders. We focused on comprehensive sources, including databases, journal articles, and reference lists from relevant publications. Our search was not limited to English language publications, ensuring a more inclusive review. The search terms employed encompassed variations of biallelic, homozygous, recessive, and *NOTCH3*. We conducted searches in prominent databases such as PubMed and ClinVar, aiming to gather information on patients with biallelic *NOTCH3* variants. The scarcity of case reports on individuals with these variants, particularly missense variants resembling CADASIL and those with loss-of-function (LoF) variants featuring severe phenotypes and childhood-onset neurodevelopmental delay, underscored the need for a more extensive patient cohort. Additionally, the inclusion of large DNA datasets worldwide enriched our understanding of these rare disorders and formed the basis for our investigation.

**Added value of this study**

Our study significantly contributes to the existing body of evidence by presenting a systematic analysis of 25 patients with biallelic variants in *NOTCH3*, encompassing 18 individuals with LoF variants and 7 with cysteine-involving missense variants. This, combined with previously published cases (25 patients, 8 LoF variants, and 17 cysteine-involving), provides a comprehensive overview of the clinical, genetic, and neuroimaging spectrum of biallelic *NOTCH3* disorders.

The key findings of our study delineate distinct disorders based on variant mechanisms. LoF variants are associated with a neurodevelopmental disorder characterised by childhood stroke, spasticity, and specific brain magnetic resonance findings. In contrast, biallelic cysteine-involving missense variants present a CADASIL phenotype, comparable in severity to patients with monoallelic variants. Notably, our study reveals that relatives carrying heterozygous LoF variants are predominantly asymptomatic, supporting a mechanism distinct from CADASIL, except for those patients with variants in the last exon (i.e. exon 33), downstream of the ANK domain, who have been associated with lateral meningocele syndrome.

Overall, our research enhances the understanding of *NOTCH3*-related disorders, offering valuable insights into their mechanisms and paving the way for improved diagnostic and therapeutic strategies.

**Implications of all the available evidence**

The implications of our study are far-reaching, particularly in the context of human health and the advancement of our understanding of *NOTCH3*-related phenotypes. *NOTCH3* variants stand as the predominant cause of hereditary brain small vessel disease, although considerable gaps persist in our knowledge regarding the pathogenesis and the broader spectrum of disorders associated with *NOTCH3*.

Our research significantly contributes to filling these gaps by delineating the entire clinical spectrum of the *NOTCH3* gene. By elucidating the different biallelic disorders related to *NOTCH3* based on variant mechanisms, our study provides a comprehensive framework. This framework serves as a crucial resource for clinicians and researchers, offering valuable insights that will facilitate a deeper understanding of these disorders. This research sets the stage for future investigations and underscores the importance of continued exploration in this critical area of neurological disorders.

## Introduction

*NOTCH3* encodes a transmembrane receptor composed of an extracellular domain formed of 34 epidermal growth factor-like repeats (EGFR) rich in cysteines, a transmembrane domain, and an intracellular domain.^1^ NOTCH3 is mainly expressed in vascular smooth muscle cells (VSMC), and it plays essential roles in different processes including the maturation of arterial vessels, injury response, VSMC differentiation, proliferation, and migration.^2^

Cerebral autosomal dominant arteriopathy with subcortical infarcts and leukoencephalopathy (CADASIL) is the most common hereditary brain small vessel disease (SVD). CADASIL is caused by heterozygous variants in *NOTCH3*,^1^^,^^3^ primarily resulting in the gain or loss of cysteine in an EGFR of the extracellular domain.^1^ The phenotype is characterised by early-onset stroke (typically between 40 and 60 years), migraine, and early dementia.^4^ Brain magnetic resonance imaging (MRI) reveals numerous lacunar infarcts and confluent white matter hyperintensities (WMH), which often extend to the anterior temporal lobes and external capsule, with or without cerebral microbleeds. Additionally, characteristic granular osmiophilic material (GOM) is observed in blood vessels both in the brain and systemically including in the skin vasculature, where it has been used as a diagnostic marker.^1^^,^^4^ While CADASIL is well-documented, there are exceptionally ultra-rare instances of individuals with biallelic *NOTCH3* variants. Some carry biallelic cysteine-involving missense variants, presenting a phenotype that falls within the CADASIL spectrum.^5^, ^6^, ^7^, ^8^, ^9^, ^10^, ^11^, ^12^, ^13^ However, the precise clinical and neuroimaging features and whether they manifest a more severe phenotype than CADASIL produced by monoallelic variants remain uncertain. Conversely, there are a few reports of patients carrying biallelic non-missense *NOTCH3* variants, particularly frameshift or premature stop-gain variants, exhibiting a neurodevelopmental phenotype with early leukoencephalopathy and stroke.^14^, ^15^, ^16^, ^17^ Nevertheless, these reports are scarce and necessitate a more extensive case study to conclusively establish their causative nature and elucidate detailed phenotypic characteristics.

Our objective is to comprehensively describe the spectrum of patients with biallelic variants in *NOTCH3* based on genetics, clinical phenotype, and neuroimaging. This involves the collection of data from 25 patients across 17 unrelated families and an extensive review of the existing literature (25 patients from 14 unrelated families).

## Methods

### Ethics

In accordance with the Declaration of Helsinki, informed consent for the publication of clinical and genetic information was obtained from the patients, their parents and/or legal guardians of all affected individuals. Ethical approval for this study was granted by the institutional review boards of University College London and the respective host institutions involved (reference numbers 19/LO/1796 and 22/NE/0080).

### Patient identification and genetics

To comprehensively delineate the phenotype of individuals with biallelic variants in *NOTCH3*, we conducted a thorough screening of DNA sequence databases from different diagnostic and research genetic laboratories worldwide. These databases included Queen Square Genomics (QSG), Centogene, GeneDx, Baylor Genetics, Invitae, 100,000 Genomes Project, GeneMatcher, Genesis, Varsome, ClinVar, ERN-Ithaca, Decipher, DDD study, Geno2MP and many other local repositories. Inclusion criterium was the presence of biallelic loss-of-function or missense pathogenic/likely pathogenic variants in *NOTCH3.* Exome sequencing and bioinformatics, followed by candidate variant Sanger sequencing and segregation analysis, were performed on DNA extracted from blood-derived leukocytes at various diagnostic and research laboratories, each following slightly different protocols.

**Fig. 1:**
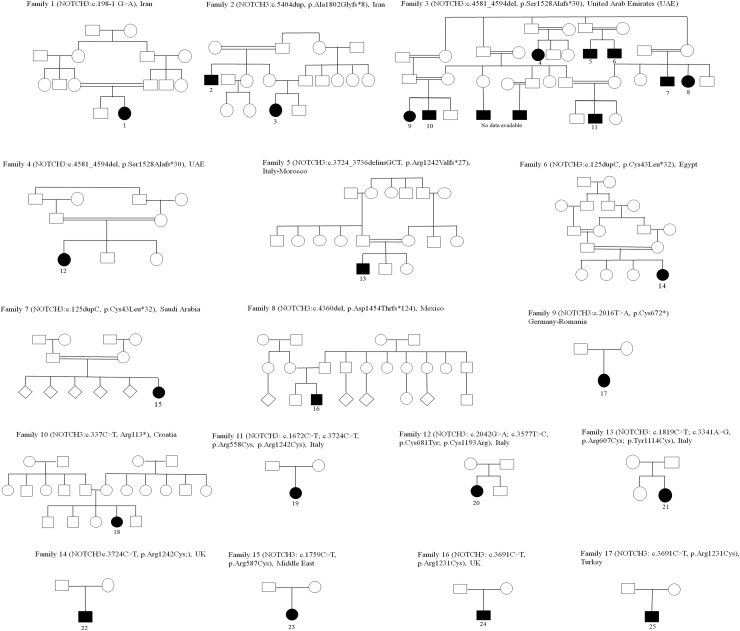
**Pedigrees of the families in the current study**.

**Fig. 2:**
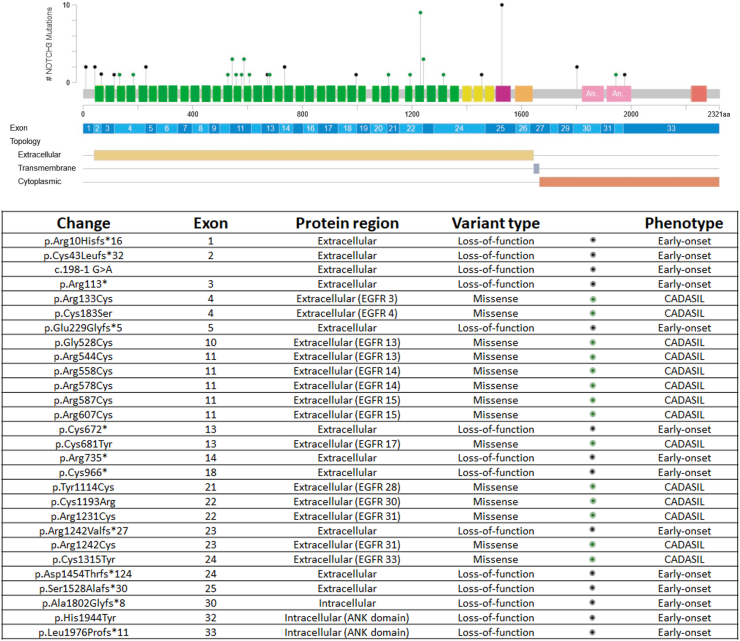
**Variants described in this study mapped to *NOTCH3* exons and domains.** The table provides details on identified variants, their effects, protein domains, and associated phenotypes. Prepared using cBioPortal (https://www.cbioportal.org/mutation_mapper). Green: EGFR domains. Yellow: LNR (Lin12 repeats), Purple and Orange: transmembrane domain, Pink: ANK domain, Red: PEST (proline, glutamic acid, serine, threonine rich) domain.

Twenty-five patients from 17 unrelated families harbouring homozygous or compound heterozygous variants in *NOTCH3* were identified and recruited for this study (P1–P25, F1–F17) (Fig. 1 and Supplementary Table S1). Among them, eighteen carried loss-of-function (LoF) variants (including one splice-site, fifteen frameshift, and two stop-gain variants) (P1–P18, F1–F10), while seven exhibited cysteine-involving missense variants (comprising two loss of cysteine and five gain of cysteine) typical of those observed in CADASIL (P19–P25, F11–F17) (Fig. 1 and Supplementary Table S1). Additionally, we conducted a systematic literature review of biallelic *NOTCH3*-SVD, identifying 13 manuscripts describing 25 patients from 14 families (P26–P50, F18–F31); eight patients from five families carrying LoF variants (P26–P33, F18–F22) and 17 patients from nine families with cysteine-involving missense variants (P34–P50, F23–F31) (Fig. 2 and Supplementary Table S1). In patients with two different variants (i.e. P19, P20, P21 and P31), they were confirmed to be in *trans* through segregation studies.

### Genetic assessment and variant analysis

*NOTCH3* variants were assessed using Varsome and Franklin by Genoox, and all were classified as likely pathogenic or pathogenic according to the American College of Medical Genetics (ACMG) criteria^18^ (Supplementary Table S1), except for three variants that were considered variants of unknown significance (VUS). Notably, two of these variants led to a cysteine-involving missense change (p.Tyr1114Cys and p. Arg1242Cys), recognised as a primary pathogenicity indicator in CADASIL.^19^^,^^20^ The third variant, present in compound heterozygosity in a published case,^14^ encoded for a non-cysteine-involving missense change in the intracellular domain of the protein (p.His1944Tyr). Frequency analysis of variants was conducted in gnomAD, UK Biobank and internal QSG databases, demonstrating their absence or extremely low frequency in the general population (Supplementary Table S1). One variant (p.Arg1231Cys) raised concerns about its pathogenicity due to its high frequency in control populations (0.489% in South Asian and 0.347% in Middle Eastern, with at least 3 homozygous carriers in gnomAD v4.0.0). This variant, generating a cysteine change, was identified in three affected families, one of which was previously published.^12^

### Clinical and neuroimaging data collection and statistics

Detailed clinical data, including biological sex, were gathered from both published and unpublished patients using a standardised clinical form (Supplementary Table S2 and Supplementary Table S3). Brain magnetic resonance imaging (MRI) scans from 41 patients and one computed tomography were reviewed by an experienced paediatric neuroradiologist (CAPFA) (Supplementary Tables S4 and S5).

Additionally, we assessed the potential difference in disease severity between individuals with biallelic *NOTCH3* variants and those with heterozygous *NOTCH3* cysteine-changing variants presenting with typical CADASIL. Our primary outcome measure for this analysis was the volume of white matter hyperintensities (WMH) observed in magnetic resonance imaging (MRI). MRI scans were accessible for three patients with homozygous cysteine-changing variants and additional two with compound heterozygous cysteine-changing variants. These were compared with a cohort of 251 patients with heterozygous cysteine-changing *NOTCH3* variants from the Cambridge CADASIL cohort.^21^ WMH were defined as increased areas of signal on fluid-attenuated inversion recovery (FLAIR) images, identified by a trained rater using a semi-automated contouring tool in the Jim analysis software (Version 8, Xinapse Systems, https://www.xinapse.com/j-im-8-software/). WMH volumes were corrected for skull size. WMH volumes between homozygous and heterozygous cases were then compared by logistic regression controlling for age, sex and EGF repeat number (≤6 or >6), as EGF position has been shown to influence disease severity.^22^

### Role of funders

Funders gave support for genetic studies and researchers’ salaries. The funders had no role in the study design, data collection, data analyses, interpretation, or writing of the report.

## Results

We identified a cohort of 50 patients with biallelic variants in *NOTCH3*, comprising 25 unreported individuals from 17 families (P1–P25, F1–F17) and 25 patients from 14 families already documented in published literature (P26–P50, F18–F31) (Supplementary Tables S1–S5).

All the patients of whom neuroimaging was available (46/50) present with brain small vessel disease and leukoencephalopathy, characteristic hallmarks of *NOTCH3*-SVD. However, a discriminating classification was undertaken, stratifying patients into two distinct groups based on variant effect, clinical phenotype, and neuroimaging findings: those with LoF variants and those with biallelic cysteine-involving missense variants.

### Patients carrying biallelic loss-of-function variants

Patients harbouring biallelic LoF variants were identified, comprising 18 affected individuals from 10 families with unpublished data (P1–P18, F1–F10) (Fig. 1) and 8 patients from 5 families previously reported in case-reports (P26–P33, F18–F22).^14^, ^15^, ^16^, ^17^ The clinical and neuroimaging data was similar between published and unpublished patients (Supplementary Table S6). Among these patients, 14 distinct variants were identified, some shared across families (Figs. 1 and 2, Supplementary Table S1). These variants included one splice-site variant causing a frameshift in coding DNA, four stop-gain variants, eight frameshift variants, and a cysteine-sparing missense variant involving the intracellular *NOTCH3* domain in compound heterozygosity with a frameshift variant (Fig. 2, Supplementary Table S1, Supplementary Table S2).

Affected individuals with biallelic LoF variants originated from different countries worldwide, primarily from the Middle East (7 families), Europe (5 families), Mexico (1 family), and Morocco (1 family). Consanguinity was prevalent in affected families (88%, 22/25), and five families exhibited more than one affected patient (F2, F3, F19, F20, and F22). Seventeen patients were female (65%), and no relevant sex-based differences were identified (Supplementary Table S7). Mean age at the onset of symptoms was 28 months, ranging from congenital to 17 years old. Symptoms at onset varied, with a predominant association with delayed motor milestones and learning difficulties (31%, 8/26), stroke-related symptoms (23%, 6/26), seizures (12%, 3/26), or hypotonia (12%, 3/26) (Supplementary Table S2).

Gestational data were available for 17 patients, with the majority being at term (82%, 14/17), and three patients being slightly preterm (35–36 weeks). Birth weight, measured in fifteen patients, revealed six with low birth weight (40%). Head circumference at birth, available in 10 patients, indicated microcephaly in only one (below percentile 2), but this proportion increased during development, with 32% of patients (6/19) showing microcephaly at the last examination.

**Fig. 3:**
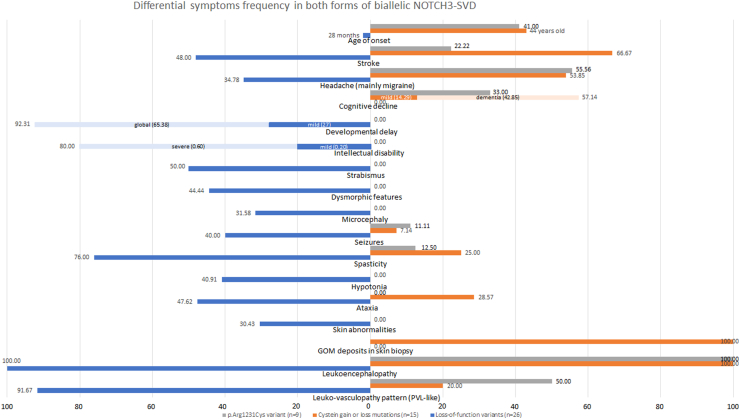
**Clinical characteristics of patients with biallelicloss-of-functionand cysteine-involving missense variants.** Graph illustrating the differential frequency of clinical and neuroimaging features among patients with biallelic loss-of-function (blue), biallelic p. Arg1231Cys (grey), and other cysteine-involving missense mutations (orange).

Developmental delay was a prevalent feature (92%, 24/26), ranging from mild or only motor delay (27%, 7/26) to global developmental impairment (65%, 17/26) (Fig. 3). Intellectual disability was observed in 81% of patients (21/26), predominantly severe, but mild in five patients. Behavioural abnormalities were noted in 35% of patients (9/26), mainly related with irritability and pseudobulbar affect (Supplementary Table S2). Speech was absent or poor in five patients (20%, 3/25), delayed in three (12%, 3/25), and present in 76% (19/25) (Table 1, Supplementary Table S2). Dysmorphic features were identified in 44% (8/18), characterised by coarse facial features, prominent eyebrows (including synophrys in one case), narrow palpebral fissure, and micrognathia.

**Table 1:** Clinical and neuroimaging data of patients with LoF variants.

Patients with loss-of-function variants (n = 26)	Number (total)	Percentage
Age of onset	28 months (congenital–17 year old)	
Birth at term	14 (17)	82.35
Low weight at birth	6 (15)	40.00
Stroke	12 (25)	48.00
Headache	8 (23)	34.78
Developmental delay	24 (26)	92.31
Motor	7 (24)	29.17
Global	17 (24)	70.83
Intellectual disability	20 (25)	80.00
Mild	5 (20)	20.00
Severe	15 (20)	80.00
Seizures	10 (25)	40.00
Spasticity	19 (25)	76.00
Ataxia	10 (21)	47.62
Strabismus	9 (18)	50.00
Sensorineural hearing loss	3 (24)	12.50
Dysmorphic features	8 (18)	44.44
Microcephaly	6 (19)	31.58
Skin abnormalities	7 (23)	30.43
GOM deposits in skin biopsy	0 (3)	0.00
Leukoencephalopathy	24 (24)	100.00
Leuko-vasculopathy pattern (PVL-like)	22 (24)	91.67
Perivascular extension of WM lesions (sunburst appearance)	21 (21)	100.00
Lateral Ventricles enlarged	20 (23)	86.96
Basal ganglia and external capsule involvement	20 (23)	86.96
Brainstem involvement	8 (21)	38.10
Hemorrhage or calcification	13 (19)	68.42
Abnormal EEG	9 (14)	64.29
Background slowing	3 (9)	33.33
Epileptic activity	6 (9)	66.67

Most patients exhibited spasticity (76%, 19/25), with either hemicorporeal or spastic quadriparesis. Stroke or transient ischemic attack occurred in 48% of cases (12/25), mainly in childhood, but also even prenatal. Seizures were present in 40% (10/25), primarily fever-related and tonic-clonic seizures. Other frequent features included hyperreflexia (91%, 21/23), strabismus (50%, 9/18), ataxia (48%, 10/21), dysarthria (39%, 7/18), and headache (35%, 8/23), although it could not be conclusively determined if the latter corresponded to migraine, a known feature of *NOTCH3*-SVD (Table 1, Fig. 3, Supplementary Table S2).

Skin abnormalities were present in seven patients with LoF variants (30%, 7/23), mostly nonspecific, but compatible with livedo reticularis in four cases. Conversely, sensorineural hearing loss (13%, 3/24) and visual impairment (19%, 4/21) were not commonly observed. Skin biopsy was performed in three patients and showed no granular osmiophilic material (GOM) deposits, typically associated with CADASIL. EEG studies were abnormal in 64% of patients (9/14), demonstrating background slowing in three patients and multifocal epileptiform activity in the remaining six.

Disease progression and severity varied among patients, with a mean age at report of 19 years old and a median of 12 years old (range, 2–49). Those with mild or only motor delay reached adulthood with independent lives, though some exhibited residual paresis and spasticity likely related to stroke; for instance, one patient did not show developmental delay and was diagnosed at 17 years old. In contrast, patients with severe global developmental delay remained severely affected with spastic quadriparesis and absent speech. No clear correlation was found between severity and specific variants, and intra-familial variability was evident, as seen in families 1 and 3 (Fig. 1, Supplementary Table S2).

**Fig. 4:**
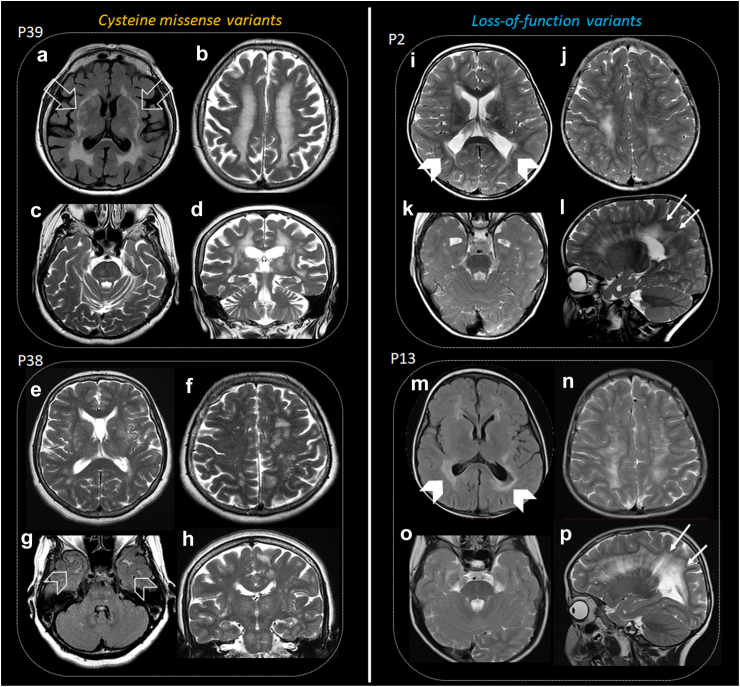
**Neuroimaging features in biallelic NOTCH3-SVD.** Axial FLAIR and T2-weighted imaging in patients with cysteine-involving missense (a-d, e-h) and loss-of-function (i-l, m-p) variants. Patient 39 exhibits extensive deep white matter involvement (b), affecting external capsules (open arrows, a), thalamus, and brainstem (c, d). Patient 38 shows fewer lesions, more prominent on the left hemisphere (f), involving temporal poles (open arrowheads, g), with mild basal ganglia, brainstem, and thalamic involvement (e, g, h). Patients 2 and 13 display pronounced white matter volume loss in posterior peritrigonal regions (squared-shape; arrowheads, i, m) with FLAIR signal intensity saturation (arrowhead, m), resembling periventricular leukomalacia. White matter lesions in both patients are predominantly periventricular, with linear perivascular extension (arrows, l, p). Brainstems are relatively spared in both patients (k, o).

From a neuroimaging perspective, patients with LoF variants typically displayed early-onset leukoencephalopathy with significant periatrial white matter volume loss (78%, 18/23), regardless of the degree of well-defined white matter lesions (Fig. 4, Supplementary Table S4). This finding appeared to be a hallmark of the disease, resulting in compensatory dilatation and deformity of the bodies and trigones of the lateral ventricles (92%, 22/24), common features described in premature newborns with white matter injury (periventricular leukomalacia). Additionally, the white matter lesions tended to have a predominant periventricular distribution, featuring linear perivascular extension (sunburst appearance) in the peritrigonal regions and corona radiata (100%, 21/21), often intermingled with mildly dilated perivascular spaces and microbleed foci (68%, 13/19), reinforcing phenotype features of leukovasculopathy and small vessel disease (Fig. 4). Moreover, basal ganglia and external capsule were often involved (87%, 20/23), while the brainstem (38%, 8/21), superficial white matter (31%, 4/13), corpus callosum (19%, 4/21), or cerebellum (6%, 1/17) were less frequently affected (Table 1, Supplementary Table S4). Notably, middle-size arteries were occasionally impaired, with one patient showing aneurysms in middle cerebral arteries, and two presenting with stenosis of intracranial carotid arteries and middle cerebral arteries. Furthermore, one patient exhibited peripheral middle-size vasculopathy manifested as cool limbs, confirmed by computed tomography. Another patient displayed grey matter subependymal heterotopia, an uncommon feature in brain small vessel disorders.

We obtained clinical data from 36 relatives carrying heterozygous LoF variants, who did not show neurological symptoms, with the exception of headache in five parents (14%, 5/36), three of them compatible with migraine. Six of these carriers of LoF variants, including four with headache, had nonspecific brain MRI, and one showed white matter brain lesions in the context of multiple sclerosis (with positive oligoclonal bands and fulfilling McDonald's criteria for the diagnosis of multiple sclerosis).^14^ The mean age at the last examination of heterozygous carriers was 47.5 years old (median 43, range from 32 to 79 years old).

### Patients with biallelic cysteine-involving missense variants

Twenty-four patients from 17 unrelated families carrying biallelic cysteine-involving missense variants were identified. This group included seven cases from seven families not previously reported (P19–P25, F11–F17) and 17 cases from nine families from prior publications (P34–P50, F23–F31).^5^, ^6^, ^7^, ^8^, ^9^, ^10^, ^11^, ^12^, ^13^ Patients not previously published generally show a milder phenotype than those reported (Supplementary Table S6). Among these patients, fourteen distinct variants were identified, with eight in homozygous state, five in compound heterozygous states, and one in both homozygous and compound heterozygous states across different individuals. Only two variants were located in EGFR domains 1–6, which has been related to a more severe phenotype.^23^ The identified variants comprised four cysteine-loss mutations, and ten cysteine-gain mutations (Fig. 2, Supplementary Table S1, Supplementary Table S3).

Nine cases (P24, P25, P44–P50) carried the variant c.3691C > T (p.Arg1231Cys), which presents some conflicting information regarding its pathogenicity. While it is present at a relatively high frequency in the common population (0.489% in South Asian, 0.347% in Middle Eastern, and three homozygous carriers in gnomAD v4.0.0), classifying it as likely benign using ACMG criteria, it produces a cysteine-involving variant. Current evidence suggests that all these variants are pathogenic, although variable penetrance might be present.^19^^,^^20^ Consequently, we decided to analyse these patients separately. The mean age of this group at the last examination was 40 years (range, 22–59), and the mean age at onset was 41 years old (range, 35–45). One-third (3/9) remained asymptomatic at the last examination. The most common symptom was migraine with or without aura (56%, 5/9), which was the only clinical finding in three of them. Three patients presented with a more severe phenotype, displaying dementia, and two of them also experienced early stroke. Brain MRI showed white matter leukoencephalopathy, which varied significantly between individuals, being confluent and severe in the two most affected patients (Table 2).

**Table 2:** Clinical and neuroimaging data of patients with cysteine-involving variants in NOTCH3 EGFr.

	p.Arg1231Cys patients (n = 9)		Remaining missense-cysteine variants (n = 15)	
	Number (total)	Percentage	Number (total)	Percentage
Age at onset	41 years old (35–53)		43 years old (13–64)	
Cognitive impairment	3 (9)	33.33	8 (14)	57.14
Developmental delay	0 (9)	0.00	0 (14)	0.00
Behavioral abnormalities	0 (7)	0.00	2 (14)	14.29
Visual impairment	2 (8)	25.00	0 (9)	0.00
Sensorineural hearing loss	0 (8)	0.00	0 (10)	0.00
Seizures	1 (9)	11.11	1 (14)	7.14
Headache	5 (9)	55.56	7 (13)	53.85
Stroke	2 (9)	22.22	10 (15)	66.67
Ataxia	0 (8)	0.00	4 (14)	28.57
Spasticity	1 (8)	12.50	3 (12)	25.00
Dysarthria	2 (8)	25.00	5 (10)	50.00
GOM	N/A	N/A	5 (5)	100.00
Leukoencephalopathy	7 (7)	100.00	15 (15)	100.00
Leukovasculopathy pattern (PVL-like)	1 (2)	50.00	2 (10)	20.00
Perivascular extension of WM lesions (sunburst appearance) (8/10)	0 (1)	0.00	8 (9)	88.89
Lateral Ventricles enlarged	1 (3)	33.33	6 (10)	60.00
Basal ganglia and external capsule involvement	1 (2)	50.00	11 (11)	100.00
Brainstem involvement	1 (2)	50.00	8 (10)	80.00
Hemorrhage or calcification	0 (1)	0.00	1 (3)	33.33
Superficial WM (T2WI) (4/14)	1 (3)	33.33	3 (11)	27.27
Abnormal EEG	0 (6)	0.00	1 (9)	11.11

The remaining 15 patients (P19–P23, P34–P43) were mostly females (60%, 9/15), and most of them were of European ancestry (60%, 9/15), followed by Chinese (27%, 4/15). In 44% of families, consanguinity was reported (4/9). The mean age at last examination was 61 years old (median 62, range, 44–75), and the mean age at the first symptoms was 43 years old (median 44, range 13–64). First symptoms at onset were mainly related to stroke (60%, 9/15) and migraine (27%, 4/15). One patient carrying the variant p.Arg1242Cys remained asymptomatic at last examination, at 47 years old. Neurodevelopment was normal in all patients, and cognitive decline was present in 57% (8/14), mostly in the form of dementia (43%, 6/14), while two patients had mild cognitive impairment. Other core CADASIL features like stroke (67%, 10/15), and headache (54%, 7/13) were also common in patients with biallelic cysteine-involving variants. Spasticity was present in 25% of patients (3/12), and ataxia in 29% of cases (4/14). Seizures were rare (7%, 1/14). Additionally, granular osmiophilic material (GOM), another feature of CADASIL, was found in all the skin biopsies performed in patients carrying biallelic cysteine variants (5/5, 100%) (Table 2).

Neuroimaging of patients with biallelic cysteine-involving missense variants was characterised by features similar to CADASIL, including confluent deep, subcortical white matter lesions (100%, 21/21) with perivascular extension (sunburst appearance) (80%, 8/10), and lesions involving the anterior temporal lobe, the basal ganglia with extension to the external capsule (92%, 12/13), and brainstem (75%, 9/12) (Table 2, Fig. 4). Periventricular leukomalacia-like appearance was less commonly present (25%, 3/12), and corpus callosum (27%, 3/11) and cerebellum (9%, 1/11) impairment were rare.

Heterozygous patients within these families commonly presented with CADASIL, although the manifestation was significantly variable and, in some families, no other relatives exhibited symptoms apart from the index cases (F11, F14, and F24). Clinical severity between homozygous and heterozygous carriers within the same family showed variability, with instances of similar or even more severe symptoms (Supplementary Table S3).

**Fig. 5:**
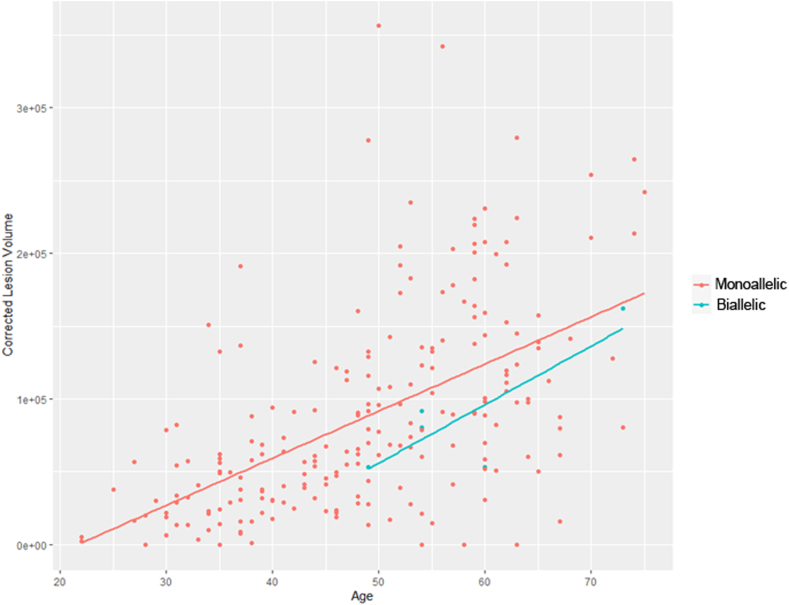
**White matter hyperintensities volume comparison between patients with biallelic cysteine-involving missense variants (n** = **5) and monoallelic CADASIL controls (n** = **251).** Graph shows that patients with biallelic variants (blue) did not exhibit larger white matter lesion volumes than patients with monoallelic CADASIL (red).

To quantitatively compare disease severity in biallelic cysteine-involving missense patients with CADASIL, we analysed white matter hyperintensities (WMH) volume in MRI scans from biallelic patients and compared them with a cohort of patients with CADASIL. The WMH volume did not differ significantly between biallelic cysteine-changing mutation patients (N = 5, including three homozygous cases, one carrying the p. Arg1231Cys variant, and two compound heterozygous cases) and heterozygous cases (N = 251) (p = 0.31 after controlling for age and sex, and p = 0.47 after also controlling for EGFR position, [logistic regression]). The distribution of WMH volumes and the best fit lines are illustrated in Fig. 5. Similar results were observed when considering only the homozygous cases.

### Genotype-phenotype correlations unveil clinical and neuroimaging disparities

While our results indicate that biallelic LoF and missense variants represent distinct disorders based on variant mechanisms, clinical phenotype, and neuroimaging (Figs. 3 and 4), both share common features associated with *NOTCH3* function and brain SVD. Common features include leukovasculopathy with predominant subcortical involvement, as well as the presence of headache and stroke (with an earlier onset in patients with LoF disease). Additionally, both groups exhibit haemorrhage or acute ischemic insults on MRI in similar proportions.

However, notable differences exist, such as the age of onset, which is earlier in patients with LoF variants (28 months vs. 44 years old). Patients with LoF variants present symptoms related to developmental and childhood disorders, including neurodevelopmental delay, intellectual disability, dysmorphic features, strabismus, hypotonia, or microcephaly. In contrast, biallelic cysteine-involving missense carriers exhibit features in adulthood, including cognitive decline and dementia. Furthermore, the presence of granular osmiophilic material (GOM) deposits is exclusive to patients with biallelic cysteine-involving missense variants, supporting distinct mechanisms in both disorders.

In terms of neuroimaging, patients with LoF variants display unique microvascular white matter lesions with periventricular leukomalacia-like features, differing from the CADASIL neuroimaging appearance observed in patients with biallelic cysteine-involving missense variants.

## Discussion

**Fig. 6:**
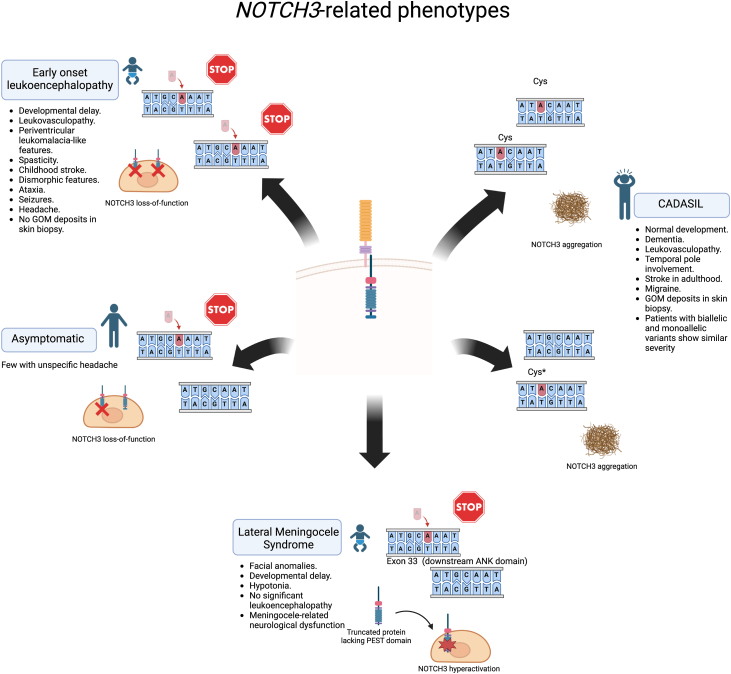
**Summary of *NOTCH3*-related disorders**. Both monoallelic and biallelic missense mutations causing gain or loss of cysteine lead to CADASIL. Few cysteine-sparing missense variants have also been reported and linked to CADASIL. Biallelic loss-of-function variants are associated with severe phenotypes, including neurodevelopmental disorders, early leukoencephalopathy, and white matter volume loss. Monoallelic carriers of these variants remain asymptomatic. However, monoallelic truncating variants in the last exon, 33, downstream of the ANK domain, are thought to produce a truncated protein lacking the PEST domain and to cause increased NOTCH3 signalling; these variants are linked to lateral meningocele syndrome. Created using BioRender (https://app.biorender.com/). GOM: granular osmiophilic material.

In this study, we present a comprehensive series of patients with biallelic variants in *NOTCH3*, contributing significant insights into its neuroradiological characteristics. Differences between LoF and cysteine-involving missense variants provide evidence supporting the genotype-phenotype variability of *NOTCH3*-SVD, all of which are characterised by small vessel involvement in the brain leading to leukoencephalopathy and subcortical strokes. We propose a classification for *NOTCH3*-SVD based on mono or biallelic variants and the type of variant (Fig. 6). Individuals with monoallelic LoF variants are either asymptomatic carriers or exhibit nonspecific headache, occasionally meeting migraine criteria. Conversely, those with biallelic LoF variants present a severe early-onset leukovasculopathy, often accompanied by developmental delay, spasticity, and childhood stroke. Patients with monoallelic gain or loss cysteine-involving missense variants typically have CADASIL, while biallelic patients manifest a similar phenotype, including adult-onset vascular leukoencephalopathy and dementia, not significantly differing from monoallelic carriers in WMH volume. Regarding the relation between cysteine-sparing missense variants and SVD it exists some controversy,^24^ although current evidence suggests that, at least some of these variants, could cause CADASIL mainly based on phenotype, neuroimaging, presence of GOM deposits, and pro-aggregation features, which are similar to those found in cysteine-involving missense variants.^25^^,^^26^ Lastly, patients with *de novo* heterozygous frameshift variants in exon 33, downstream of the ANK domain, have been associated with Lateral Meningocele Syndrome (LMS). These variants are thought to produce a truncated protein lacking PEST domain and to induce hyperactivity of the intracellular domain of *NOTCH3*.^27^ LMS is characterised by multiple lateral spinal meningoceles, distinctive craniofacial characteristics, joint hyperextensibility, hypotonia, and anomalies in the skeletal, cardiac, and urogenital systems.^27^^,^^28^ Significantly, leukoencephalopathy is not a hallmark of LMS.^28^ Moreover, one of the previously published patients with a LoF phenotype has a variant in exon 33 affecting ANK domain,^14^ which suggests that variants in this domain (although predicted to avoid nonsense-mediated decay) may cause a LoF effect (Supplementary Figure S1).

Notably, variable interindividual expressivity is common within both missense and LoF variants groups, a phenomenon also observed in CADASIL. Despite recent suggestions of the influence of variant position,^19^^,^^29^ variable expressivity is identified even in patients carrying the same variants within the same family (as observed in families F2, F3, or F31), indicating that other unexplored factors may regulate the phenotype.^30^ An important example of this broad variability is the presence of missense cysteine-involving variants in population cohorts^31^^,^^32^ suggesting reduced penetrance of some of these variants, although these patients present with a higher risk of stroke and dementia.^23^ Hence, the variant p.Arg1231Cys is relatively common in South Asian (0.489%) and Middle Eastern (0.347%) and is probably more benign due to, at least partially, its EGFR domain location.^29^ However, further research will be needed to completely validate this variant pathogenicity and the reason it is so frequent, especially in some populations, which might be related to a founder effect or other genetic modulators.

Our findings enhance the understanding of CADASIL pathogenesis, where a consensus on whether this results from a gain or loss of NOTCH3 function is yet to be established. NOTCH3 haploinsufficiency alone is insufficient to produce CADASIL symptoms, as demonstrated by the mainly asymptomatic carriers of LoF variants in the families described here, consistent with similar observations in previously published reports.^33^^,^^34^ This finding supports the hypothesis of toxic aggregated granular osmiophilic material secondary to misfolded proteins due to missense cysteine-involving mutations. Animal models aid in elucidating the differences between these disorders. Indeed, mouse models carrying both null and cysteine-involving variants have been developed.^35^^,^^36^ While null Notch3 mice don't show aggregates and are viable and fertile,^37^ they present with abnormal response to pressure in brain and tail arteries.^38^ On the other hand, mice carrying cysteine-involving variants usually present with aggregates that include wildtype Notch3 and smooth muscle cell defects.^39^ These studies support the gain-of-toxicity hypothesis in CADASIL^39^ and wildtype Notch3 aggregation in these mice may explain why biallelic cysteine variants do not generate a more severe phenotype, as both alleles would be aggregated in both scenarios. Conversely, the relatively benign features of null mice confront the severe phenotype of patients with biallelic LoF variants.

One limitation of this study is the lack of brain MRIs from most asymptomatic individuals carrying monoallelic LoF variants, potentially revealing clinically silent white matter lesions. Although the available MRIs (n = 6) were normal or showed mild incidental foci of white matter lesions with no clear significance,^16^ this highlights an area for future investigation. Similarly, we lack neuroimaging data from 4 patients with biallelic variants, one carrying a LoF variant (P10) and three with cysteine variants (P22, P45 and P48). However, as most carried known variants present in other patients (P10, P45, and P48) and in most cases cysteine-related (P22, P45, and P48) we included the clinical data in our analyses. Furthermore, the absence of a more severe phenotype in biallelic missense patients with a “double hit” suggests that NOTCH3 aggregation might cause disease in a dose-independent manner, which could have implications for treatment strategies.

Patients with *NOTCH3* LoF variants exhibit leukovasculopathy with periventricular leukomalacia-like patterns in neuroimaging, which are likely related to brain small vessel injury and ischemic insult in early stages of life or even prenatal as shown in preterm hypoxic-ischemic encephalopathy,^40^ which must be considered as a differential diagnosis. Additional differential diagnoses for these patterns in the paediatric population include viral infections, such as parechovirus, certain types of pontocerebellar hypoplasia (particularly type 9–AMPD2—MIM 615809), various forms of hereditary spastic paraplegia (SPG11—MIM 604360–and SPG15—MIM 270700), and mutations in type IV collagen (COL4A1—MIM 175780–and COL4A2—MIM 614483). Other differentials encompass genetically related leuko-vasculopathy, such as cathepsin A-related arteriopathy with strokes and leukoencephalopathy (CARASAL)^41^– MIM ∗613,111 –, cerebral autosomal recessive arteriopathy with subcortical infarcts and leukoencephalopathy (CARASIL)—MIM 600142, due to variants in *HTRA1,* which has been also identified in CADASIL aggregates and presents with characteristic features such as alopecia, spondylosis, and severe leuko-vasculopathy with extensive involvement of the posterior fossa.^42^^,^^43^ Furthermore, biallelic variants in *NIT1* have been recently described to produce a disorder characterised by movement disorders, dilated perivascular spaces, and intracranial haemorrhage.^44^ Additionally, few cases of paediatric CADASIL have been reported, displaying variable and usually milder phenotypes (both clinically and radiologically) than patients with biallelic LoF variants.^45^^,^^46^ Furthermore, 44% of patients with LoF variants showed dysmorphic features, which suggest an involvement of NOTCH3 signalling in musculoskeletal development, which is also supported by the dysmorphic features and musculoskeletal abnormalities identified in lateral meningocele syndrome^27^ and the presence of dysmorphic features in other disorders related to NOTCH signalling, including Alagille syndrome caused by variants in *JAG1* and *NOTCH2* (MIM 118450 and 610205, respectively).^47^

In conclusion, *NOTCH3*-SVD is more complex than previously appreciated. This study focuses on biallelic *NOTCH3*-SVD to further elucidate the genotype-phenotype variability in patients exhibiting two distinct phenotypes likely related to different disease mechanisms.

## Contributors

RM, HH and HSM designed the study; PI, AMAS, GE, LP, DL, BPHC, AAF, FAA, JG, EF, KV, FBO, HoH, MZ, BMA, FSA, MR, LT, SB, FP, AAT, MA, AP, AC, NDS, FK, RaM, MS, GZ, LS, TBH, AR, SZ, LRG, AÇ, LO, KGH, MB, PB,WCJ, EGK,WKC,VN, FA, BS, HSM, HH and RM performed data analysis and collected the clinical data; PI and RM performed the bibliographic review; CAPFA, LP and AJ performed the neuroimaging analyses; PI, CAPFA and RM wrote the draft. PI and RM have accessed and verified the underlying raw data. All authors revised and approved the manuscript.

## Data sharing statement

Raw data of clinical features, neuroimaging and statistical analyses will be available upon reasonable request to the corresponding author.

## Declaration of interests

Wendy Chung is on the board of directors of Prime Medicine. Stephan Zuchner has received consultancy honoraria from Neurogene, AegleaBioTherapeutics, Applied Therapeutics, and is an unpaid officer of the TGP foundation, all unrelated to the present manuscript. Elisa Fazzi has received honoraria from GW Pharma. Nicola De Stefano has received honoraria from Biogen-Idec, Genzyme, Immunic, Merck, Novartis, Roche, Celgene, and Teva for consulting services, speaking, and travel support. He serves on advisory boards for Merck, Novartis, Biogen-Idec, Immunic, Roche, and Genzyme, and he has received research grant support from the Italian MS Society. Lyn R Griffiths has received grants from the Australian National Health and Medical Research Council, Variant Bio, US Department of Defense and US Migraine Research Foundation as well as honoraria from Teva, Springer Nature, and Association of Migraine Disorders and she is Board of Censors, Diagnostic Genomics Human Genetics Assoc Australia and member of the Human Genetics Australasia Advisory Board. Hugh S Markus has received peer reviewed grants from the Medical Research Council, British Heart Foundation, National Institute of Health Research, and the Alzheimer Society, and is editor in chief of the International Journal of Stroke.

## Appendix A Supplementary data

Supplementary Figure S1 **Exon 33 variants and related phenotypes.** All the variants related to lateral meningocele syndrome (LMS) to date are located in exon 33, downstream of the ANK domain while there is one variant present in a previously published patient with a loss-of-function phenotype located in exon 33 within the ANK domain (Stellingwerff et al. 2022). As ANK domain is related to NICD interaction with other proteins, variants affecting this domain may produce a loss-of-function effect rather than the gain-of-function related to LMS, where the negative regulator PEST domain is absent. Created using BioRender (https://app.biorender.com/).

Supplementary Table S1 Variants related to biallelic NOTCH3-disorders.

Supplementary Table S2 Clinical features of patients with biallelic loss-of-function variants in *NOTCH3*.

Supplementary Table S3 Clinical features of patients with biallelic cysteine-involving variants in NOTCH3 EGFR.

Supplementary Table S4 Neuroimaging features of patients with biallelic loss-of-function variants in *NOTCH3.*

Supplementary Table S5 Neuroimaging features of patients with biallelic cysteine-involving variants in NOTCH3 EGFR.

Supplementary Table S6 Comparison between reported and unreported patients.

Supplementary Table S7 Sex-disaggregated data.
